# Supplementary material for: Strengthening the Culture of Well-Being in Rural Hospitals Through RISE Peer Support
Source: Healthcare (Basel). 2025 Dec 30;14(1):91. doi: 10.3390/healthcare14010091 (PMC12786105; doi:10.3390/healthcare14010091)
Supplement: Supplementary file 1 [file healthcare-14-00091-s001.zip › healthcare-4026634-supplementary.pdf]

Supplementary File S1. Description of the survey items

Anxiety, Burnout, Resilience Questionnaire

Item	Stem	Response Scale
1	I would rate the overall well-being of people who work in this hospital as	Poor<br>Fair<br>Good<br>Very Good<br>Excellent
2	I would rate the overall resilience to stressful work-related events of people in this hospital as	Poor<br>Fair<br>Good<br>Very Good<br>Excellent
3	Overall, I am _____ with my job.	Not at all satisfied<br>Not too satisfied<br>Somewhat satisfied<br>Very satisfied
4	I can count on my supervisor for support when I need it.	Strongly disagree<br>Somewhat disagree<br>Somewhat agree<br>Strongly agree
5	I can count on my coworkers for support when I need it.	Strongly disagree<br>Somewhat disagree<br>Somewhat agree<br>Strongly agree
6	I trust the management at my organization.	Strongly disagree<br>Somewhat disagree<br>Somewhat agree<br>Strongly agree
7	My organization is committed to employee health and well-being.	Strongly disagree<br>Somewhat disagree<br>Somewhat agree<br>Strongly agree
8	To what extent do you agree or disagree with the following statements? If I experience a stressful work-related event, there is someone in my organization...<br>d) My organization provides a confidential peer support program for employees who have experienced a stressful work-related event.<br>e) I know who to contact for peer support in my organization if I experience a stressful work-related event.<br>a. I have prompt access to trained peer support staff who can help me if I experience a stressful work-related event.	Strongly disagree<br>Somewhat disagree<br>Neither agree or disagree<br>Somewhat agree<br>Strongly agree

9	To what extent do you agree or disagree with the following statements? If I experience a stressful work-related event, there is someone in my organization...<br>f. To listen to me without judgment.<br>g. To empathize with my situation.<br>h. To help me process my experience.<br>i. To provide me with emotional support.<br>j. To help me to be more resilient.	Strongly disagree<br>Somewhat disagree<br>Neither agree or disagree<br>Somewhat agree<br>Strongly agree
10	Anxiety Scale (Part I)<br>Over the last 2 weeks, how often have you been bothered by the following problems?<br>a) Feeling nervous, anxious, or on edge<br>b) Not being able to stop or control worrying<br>c) Worrying too much about different things<br>d) Trouble relaxing<br>e) Being so restless that it is hard to sit still<br>f) Becoming easily annoyed or irritable<br>g. Feeling afraid as if something awful might happen	Not at all<br>Several days<br>More than half of the days<br>Nearly every day
11	Anxiety Scale (Part II)<br>If you checked off any problems, how difficult have these problems made for you to do your work, take care of things at home, or get along with other people?	Not difficult at all<br>Somewhat difficult<br>Very difficult<br>Extremely difficult
12	Burnout Scale (Part I)<br>How often do you feel burned out from your work?	Never<br>A few times a year or less<br>Once a month or less<br>A few times a month<br>Once a week<br>A few times a week<br>Everyday
13	Burnout Scale (Part II)<br>How often do you feel you've become more callous toward people since you took this job?	Never<br>A few times a year or less<br>Once a month or less<br>A few times a month<br>Once a week<br>A few times a week<br>Everyday
14	Resilience Scale<br>For each item, please select the answer that best indicates how much you agree with the following statements as they apply to you over the last month. If a particular situation has not occurred recently, answer according to how you think you would have felt.<br>a) I am able to adapt when changes occur<br>b. I tend to bounce back after illness, injury, or other hardships	Not at all true<br>Rarely true<br>Sometimes true<br>Often true<br>True nearly all the time

CULTURE OF WELLBEING QUESTIONS

Question Number	Question	Response Choices and Numeric code	Subscale
4	I can count on my supervisor for support when I need it.	Strongly disagree- 1<br>Somewhat disagree - 2<br>Somewhat agree - 3<br>Strongly agree - 4	Organizational Support
5	I can count on my coworkers for support when I need it.	Strongly disagree- 1<br>Somewhat disagree - 2<br>Somewhat agree - 3<br>Strongly agree - 4	Organizational Support
6	I trust the management at my organization.	Strongly disagree- 1<br>Somewhat disagree - 2<br>Somewhat agree - 3<br>Strongly agree - 4	Organizational Support
7	My organization is committed to employee health and well-being.	Strongly disagree- 1<br>Somewhat disagree - 2<br>Somewhat agree - 3<br>Strongly agree - 4	Organizational Support
8	To what extent do you agree or disagree with the following statements?<br>a. My organization provides a confidential peer support program for employees who have experienced a stressful work-related event.<br>b. I know who to contact for peer support in my organization if I experience a stressful work-related event.<br>c. I have prompt access to trained peer support staff who can help me if I experience a stressful work-related event.	Strongly disagree - 1<br>Somewhat disagree - 2<br>Neither agree or disagree - 3<br>Somewhat agree - 4<br>Strongly agree - 5	Peer Support
9	To what extent do you agree or disagree with the following statements? If I experience a stressful work-related event, there is someone in my organization...<br>a. To listen to me without judgment.<br>b. To empathize with my situation.<br>c. To help me process my experience.<br>d. To provide me with emotional support.<br>e. To help me to be more resilient.	Strongly disagree - 1<br>Somewhat disagree - 2<br>Neither agree or disagree - 3<br>Somewhat agree - 4<br>Strongly agree - 5	Availability of Support

RISE Program Evaluation Questionnaire

Section 1: Awareness and Exposure

1. Have you heard of RISE?

☐ Yes ☐ No

2. How did you hear about RISE? (check all that apply)

☐ Staff meeting

☐ Word of mouth

☐ Brochure

☐ Email

☐ Poster

☐ I am a RISE responder

☐ I know a RISE responder

☐ I participated in training

☐ Other (please specify): ___________

Section 2: Utilization of RISE

3. Have you ever used RISE?

☐ Yes ☐ No

4. If you did not use RISE, why not? (check all that apply)

☐ I didn’t feel comfortable using it (please explain): ___________

☐ I didn’t know about it

☐ I didn’t know how to access it

☐ I didn’t need it

☐ I intended to use it but didn’t (please explain): ___________

☐ Other (please explain): ___________

Section 3: Satisfaction with RISE (for those who used the program)

Please indicate your level of agreement with the following statements:

RISE is easy to use

Strongly Disagree ☐ Somewhat Disagree ☐ Neither Agree nor Disagree ☐ Somewhat Agree ☐ Strongly Agree ☐

I received a prompt response from RISE

Strongly Disagree ☐ Somewhat Disagree ☐ Neither Agree nor Disagree ☐ Somewhat Agree ☐ Strongly Agree ☐

I felt comfortable using RISE

Strongly Disagree ☐ Somewhat Disagree ☐ Neither Agree nor Disagree ☐ Somewhat Agree ☐ Strongly Agree ☐

I received the support I needed

Strongly Disagree ☐ Somewhat Disagree ☐ Neither Agree nor Disagree ☐ Somewhat Agree ☐ Strongly Agree ☐

I am satisfied with my RISE experience

Strongly Disagree ☐ Somewhat Disagree ☐ Neither Agree nor Disagree ☐ Somewhat Agree ☐ Strongly Agree ☐

I would recommend RISE to colleagues

Strongly Disagree ☐ Somewhat Disagree ☐ Neither Agree nor Disagree ☐ Somewhat Agree ☐ Strongly Agree ☐

Section 4: Workplace Culture and Support

Please indicate your level of agreement with the following statements:

My supervisor encourages staff to use RISE

Strongly Disagree ☐ Somewhat Disagree ☐ Neither Agree nor Disagree ☐ Somewhat Agree ☐ Strongly Agree ☐

My coworkers encourage staff to use RISE

Strongly Disagree ☐ Somewhat Disagree ☐ Neither Agree nor Disagree ☐ Somewhat Agree ☐ Strongly Agree ☐

I would feel comfortable using RISE in the future

Strongly Disagree ☐ Somewhat Disagree ☐ Neither Agree nor Disagree ☐ Somewhat Agree ☐ Strongly Agree ☐

I would refer coworkers to RISE

Strongly Disagree ☐ Somewhat Disagree ☐ Neither Agree nor Disagree ☐ Somewhat Agree ☐ Strongly Agree ☐

My coworkers would use RISE if needed

Strongly Disagree ☐ Somewhat Disagree ☐ Neither Agree nor Disagree ☐ Somewhat Agree ☐ Strongly Agree ☐

Having RISE shows my organization is committed to staff well-being

Strongly Disagree ☐ Somewhat Disagree ☐ Neither Agree nor Disagree ☐ Somewhat Agree ☐ Strongly Agree ☐

Having RISE ensures support is available when needed

Strongly Disagree ☐ Somewhat Disagree ☐ Neither Agree nor Disagree ☐ Somewhat Agree ☐ Strongly Agree ☐

Having RISE shows my well-being matters to the organization

Strongly Disagree ☐ Somewhat Disagree ☐ Neither Agree nor Disagree ☐ Somewhat Agree ☐ Strongly Agree ☐

RISE can improve staff morale

Strongly Disagree ☐ Somewhat Disagree ☐ Neither Agree nor Disagree ☐ Somewhat Agree ☐ Strongly Agree ☐

RISE can promote a culture of well-being

Strongly Disagree ☐ Somewhat Disagree ☐ Neither Agree nor Disagree ☐ Somewhat Agree ☐ Strongly Agree ☐

RISE can foster a supportive environment

Strongly Disagree ☐ Somewhat Disagree ☐ Neither Agree nor Disagree ☐ Somewhat Agree ☐ Strongly Agree ☐

RISE can increase staff resilience

Strongly Disagree ☐ Somewhat Disagree ☐ Neither Agree nor Disagree ☐ Somewhat Agree ☐ Strongly Agree ☐

RISE can improve coping with stress

Strongly Disagree ☐ Somewhat Disagree ☐ Neither Agree nor Disagree ☐ Somewhat Agree ☐ Strongly Agree ☐

RISE can reduce burnout

Strongly Disagree ☐ Somewhat Disagree ☐ Neither Agree nor Disagree ☐ Somewhat Agree ☐ Strongly Agree ☐

RISE can reduce turnover

Strongly Disagree ☐ Somewhat Disagree ☐ Neither Agree nor Disagree ☐ Somewhat Agree ☐ Strongly Agree ☐

Section 5: Observations

I am aware of colleagues who have been traumatized at work.

☐ Yes ☐ No ☐ Unsure

I believe those colleagues received appropriate support.

☐ Yes ☐ No ☐ Unsure
